# Multilingual Virtual Healthcare Assistant

**DOI:** 10.1002/hcs2.70031

**Published:** 2025-07-31

**Authors:** Geetika Munjal, Piyush Agarwal, Lakshay Goyal, Nandy Samiran

**Affiliations:** ^1^ Amity School of Engineering and Technology Noida India

**Keywords:** BLEU score, encoder‐only transformer model, healthcare chatbot, LSTM, NLP, virtual healthcare

## Abstract

This study proposes a virtual healthcare assistant framework designed to provide support in multiple languages for efficient and accurate healthcare assistance. The system employs a transformer model to process sophisticated, multilingual user inputs and gain improved contextual understanding compared to conventional models, including long short‐term memory (LSTM) models. In contrast to LSTMs, which sequence processes information and may experience challenges with long‐range dependencies, transformers utilize self‐attention to learn relationships among every aspect of the input in parallel. This enables them to execute more accurately in various languages and contexts, making them well‐suited for applications such as translation, summarization, and conversational Comparative evaluations revealed the superiority of the transformer model (accuracy rate: 85%) compared with that of the LSTM model (accuracy rate: 65%). The experiments revealed several advantages of the transformer architecture over the LSTM model, such as more effective self‐attention, the ability for models to work in parallel with each other, and contextual understanding for better multilingual compatibility. Additionally, our prediction model exhibited effectiveness for disease diagnosis, with accuracy of 85% or greater in identifying the relationship between symptoms and diseases among different demographics. The system provides translation support from English to other languages, with conversion to French (Bilingual Evaluation Understudy score: 0.7), followed by English to Hindi (0.6). The lowest Bilingual Evaluation Understudy score was found for English to Telugu (0.39). This virtual assistant can also perform symptom analysis and disease prediction, with output given in the preferred language of the user.

## Graphical abstract

In current study a multilingual health interface is proposed that can receive a voice, text, and image message to report the symptoms individually. This tool will increase access, early diagnosis, and precision of the diagnosis on a different user base.

**Abbreviations**
AI: artificial intelligence
BERT: bidirectional encoder representations from transformers
BLEU: bilingual evaluation understudy
LSTM: long short‐term memory
NLP: natural language processing
RNN: recurrent neural network

## 1 Introduction

Artificial intelligence (AI) technologies are developing quickly, with possible applications in the healthcare sector that could potentially enable services to become more personalized, faster, and more inclusive. Virtual health assistants or AI powered software agents that aid patients and healthcare providers represent an exciting innovation in this field. Hybrid AI systems together with natural language processing (NLP), machine learning (ML) systems, and data analytics systems are used by these virtual assistants to deliver tailored health information to patients, monitor patient's conditions, and facilitate communication between patients and healthcare professionals [1]. Information, services, and materials relating to health in contemporary societies have become increasingly high‐tech and digitally accessible. Many modern healthcare services use digital technology, with increasing availability of home visits by doctors or therapists through the Internet or mobile applications, data sharing between doctors and patients through patient portals, and health meter applications as digital tools that users can access in different ways [2, 3]. These innovations can reduce the administrative burden on members of healthcare teams, allowing them to devote more of their time to patient care, which can improve patient's outcomes in the long run. Providers can spend more time and effort helping patients understand their needs, developing meaningful relationships, and providing high quality care when providers have cleared their calendars of non‐patient‐oriented work.

The term “digital divide” refers to a gap between users of information technology, especially internet services, and non‐users. Importantly, some issues are specific to certain categories of users, such as older people, individuals with disabilities, and individuals with low incomes. The digital divide aggravates and maintains health imbalances, limiting the choices of underprivileged groups in accessing suitable forms of healthcare services. In the context of the healthcare sector, this division can determine whether or not individuals receive quality healthcare services at the right time [4].

Such assistants enable individuals with low literacy to engage with technology through voice rather than typing. Patients who engage with assistants are more likely to follow treatment and preventive health plans, and to live healthier lifestyles [2]. For example, assistants can perform the following functions: reminding patients of upcoming appointments, encouraging patients to be adherent with drug regimens, and providing patients with tailor‐made health advice, on the basis of patients' individual health data [5, 6]. Engaged patients are more likely to adhere to treatment plans, participate in preventive health measures, and maintain healthier lifestyles.

Multilingual chatbot models depend critically on deep learning architecture to provide accurate and efficient NLP in multiple languages [7]. Transformer‐based architectures like Bidirectional Encoder Representations from Transformers. and GPT have been demonstrated to have the capacity to deal with sequential data and the learning of contextual information. Because self‐attention mechanisms make transformers highly effective at understanding the relations of words in a sentence, they are used for functions like language translation, sentiment analysis, and text generation [8]. This architecture is well suited to the chatbot model because it can smoothly process different languages, including languages with complicated grammar and syntax. We trained the current system on a multilingual data set to learn shared representation across languages, enabling the model to learn to understand and generate responses in different linguistic contexts. Further reducing the computational requirements is the use of pretrained models which also provide scalability to low‐resource languages such as Gujarati, Bengali, and Kannada. Considering the importance of multilingual virtual assistants, the current study proposes the Virtual Vita system, which was designed with the following objectives: (1) Improving the health experience: Creating AI‐driven recommendations within the context of users’ needs to provide better chances of patient engagement, and better adherence to treatment. (2) Improving accessibility: Offering a service that is suitable for underserved regions or languages that face barriers using NLP and real time language translation. (3) Automating routine healthcare tasks: Providing symptom tracking, medication adherence, and appointment reminders to help healthcare providers reduce their administrative workload.

## 2 Methods

The current study used the “Disease Prediction Based on Symptoms” data set hosted on Kaggle, which is mainly used to predict diseases on the basis of input symptoms [9]. The data set obtained from Kaggle includes an easy‐to‐read data base with information about diseases, symptoms, cures, specialists, and risks related to the diseases. This data set subdivides ailments like flu, bronchitis, pneumonia, heart attack, and stroke by their symptoms (fever, cough, chest pain) and by the prescription of antibiotics or an emergency call. Every disease is then linked to the correct disease management specialists, such as general practitioners, respiratory physicians, cardiologists, or neurologists, based on the risks indicating low through to high severity. This data set finds its utility in most diagnosis and prognosis models and real‐ time decision making in the healthcare sector [10, 11].

Diversity of demography was evaluated to determine the effectiveness of our Disease Diagnosis Data set [12] for symptom‐disease prediction, and the data set was thoroughly tested to examine different age groups, genders, and patient attributes. This data set is valuable because of its comprehensive inclusion of symptoms, sensor readings, and diagnoses, which can provide a solid foundation for AI‐driven disease prediction models [12, 13].

**Figure 1:**
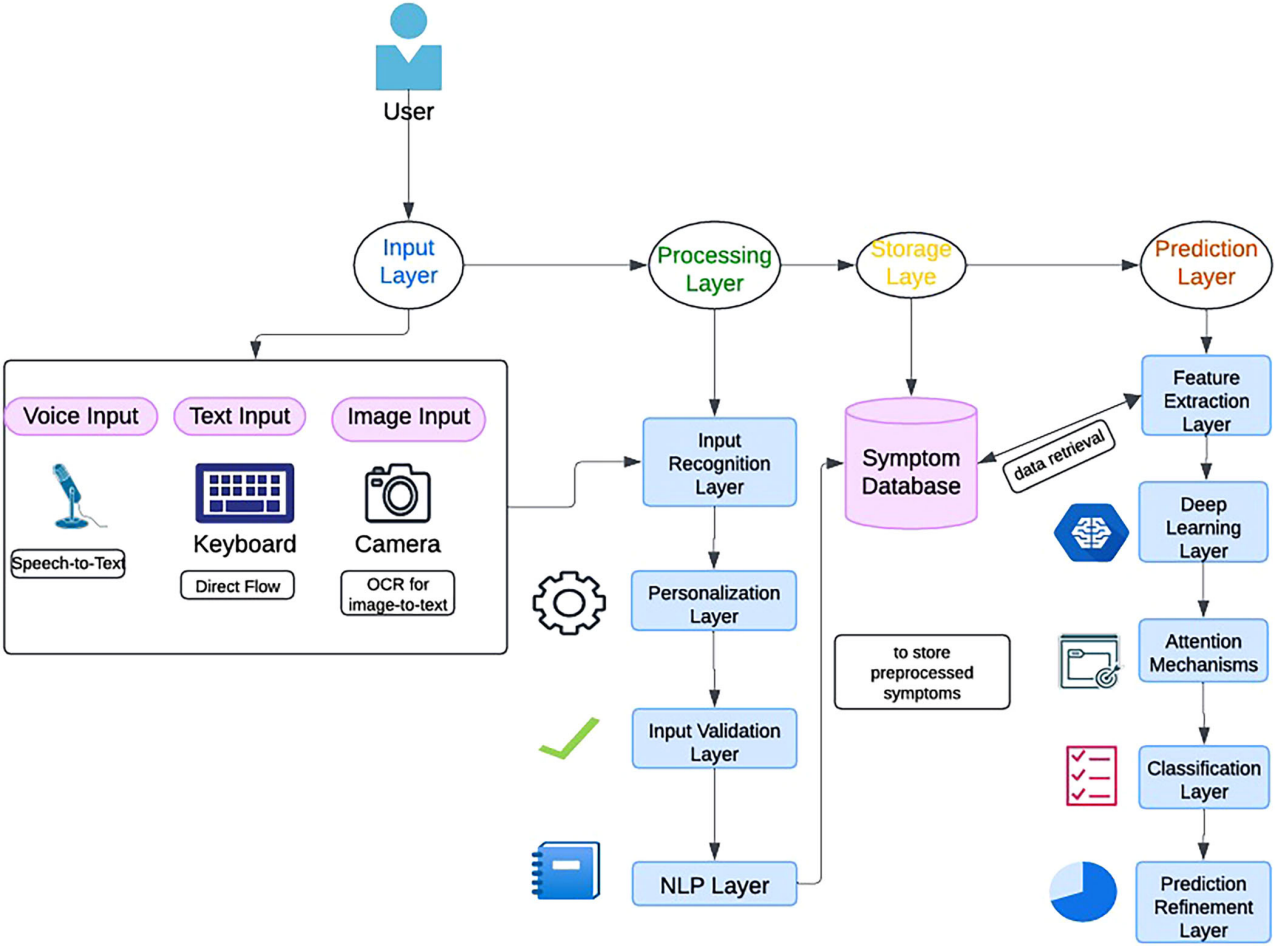
Proposed framework for healthcare assistant virtual vita.

The methodology underlying the Virtual Vita platform was designed to provide a robust framework for disease prediction and personalized education through advanced AI techniques. The steps followed in the current study are outlined in Figure 1. In preprocessing, missing and irrelevant entries were removed, a custom built vocabulary was used to tokenize symptom descriptions and convert them into numerical values. Padding and truncation were performed to construct a fixed sequence. Finally, the data set was split into training data, validation data, and test data to develop proper evaluation model. Inputs from text were converted to a structured form (e.g., tokens) then further encoded and stored for future comparison. Once preprocessing was finished, the data were appropriately structured to begin training the disease prediction model [14].

As can be seen in Figure 1, the input layer takes input from users with varied needs, supporting voice, text, and image input. This input is processed by processing layer to further feed to the system. The input recognition layer converts user inputs into machine readable formats (e.g., speech). Personalization layer adaptation is based on user profiles, preferences, and accessibility, and its output provides the user with access to input their symptoms. In the next step, the user reports symptoms through the interface, using text and voice explain the symptoms and display an image with a query, creating an effort with a wait, and providing a test [15].

In symptom translation, the symptoms are provided by users in natural language followed by tokenization, lemmatization, and entity reorganization referring to medical terms. Feature normalization is performed to standardize symptom reporting across different languages and medical practices. These symptoms are mapped to standard codes (e.g., Systematized Nomenclature of Medicine—Clinical Terms or International Classification of Diseases, standards diagnosis codes that can help in prediction) [16]. Disease prediction identifies early symptoms and predicts possible diseases by extraction features in the form of key patterns and correlation with symptoms [17]. As part of the learning model for attention, classification and prediction refinement help in mapping to disease probabilities and ranking the disease likelihood, giving further insight including risk factors or severity [18].

**Figure 2:**
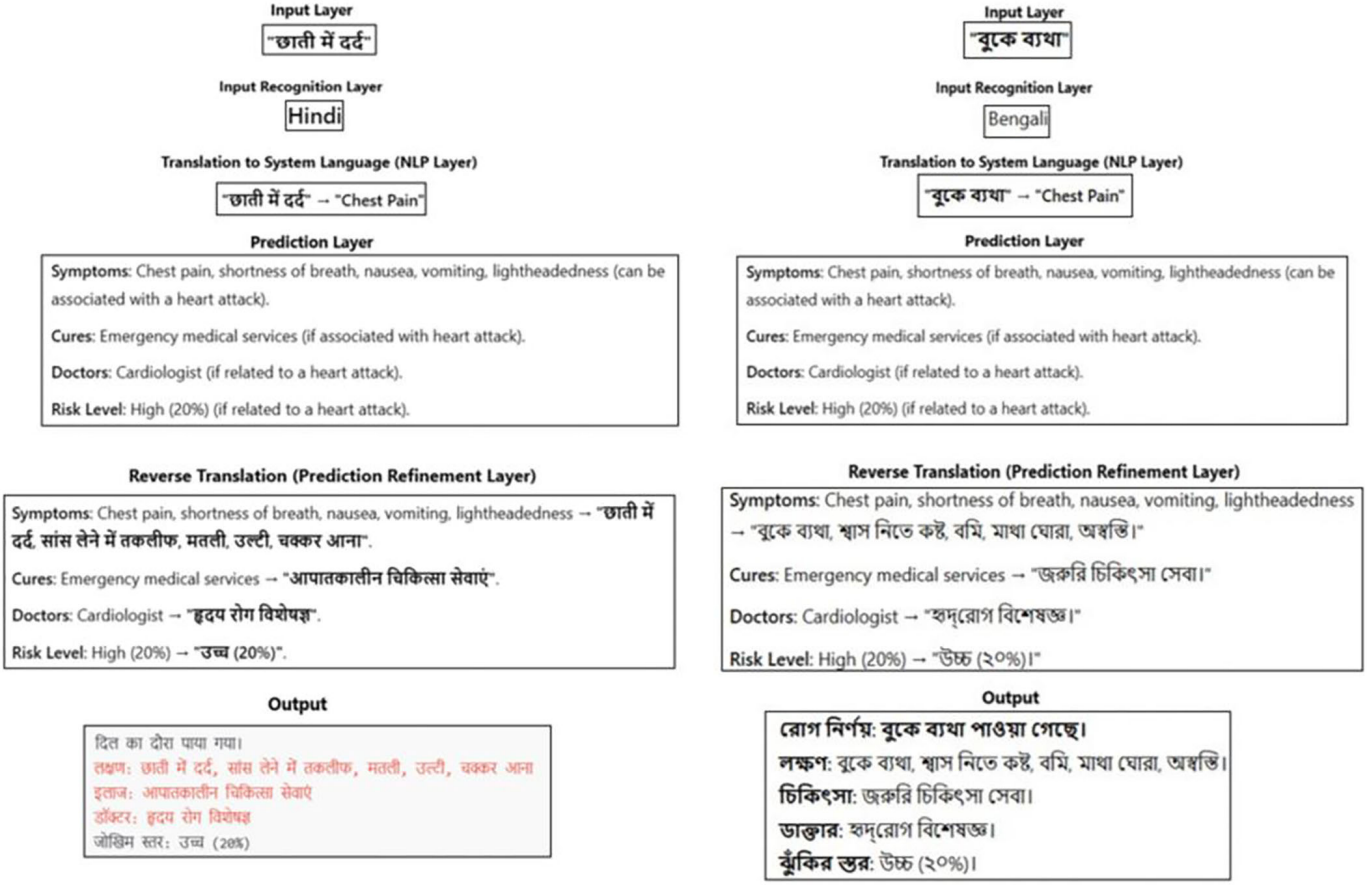
Multilingual chabot workflow.

Multilingual chatbot models perform an important role of bridging gaps in language, which enables smooth conversation with users. User input can be in different regional languages, which the system receives and processes to the working system language (e.g., English) as shown in Figure 2. The proposed multilingual chatbot includes NLP and real time translation to provide personalized, accessible health advice and information [19]. This system leverages the power of the Google Web Speech Application Programming Interface for speech‐to‐text capabilities and the Google Translate Application Programming Interface for seamless language translation to aid language detection, translation from and to multiple languages, and context aware symptom and condition prediction. The process of this comprehensive strategy was designed to increase user engagement and understanding but also to help healthcare practitioners utilize digital technologies to improve care quality and patient satisfaction, leading to the wider utilization of these technologies in healthcare settings in the future [20]. A snapshot of a conversation using the system is shown in Figure 2, where the input undergoes translation from a regional language, such as Hindi or Bengali. The input language is preprocessed by translating the input language into English, then fed into the prediction phase to predict a treatment (alongside a comparatively simple associated risk). The output in this layer is generated in English but is reverse translated to Hindi or Bengali and shown back in the output layer [21].

The encoder‐only transformer architecture is used for NLP and multilingual text analysis. Input embedding: raw input such as symptoms or voice is mapped to perform input embedding. Positional encoding: positional context is added to embedded input to address the lack of positional information inherent in transformers. The self‐attention mechanism calculates attention scores to focus on useful contextual features while multi head attention interprets better contextual understanding. Residual connections and layer normalization are used with feedforward layers to further leverage a feature extractor for effortless and stable training [16]. Context‐aware representations are then generated through the encoder‐decoder structure for predictions [22]. The feature extraction layer relies heavily on transformers, which take inputs and transform them into semantically rich embeddings. Attention mechanisms are used to analyze symptoms and find patterns with the deep learning layer. The prediction refinement layer receives feedback to better handle errors and make predictions closer to those intended by the user [9].

## 3 Results

**Figure 3:**
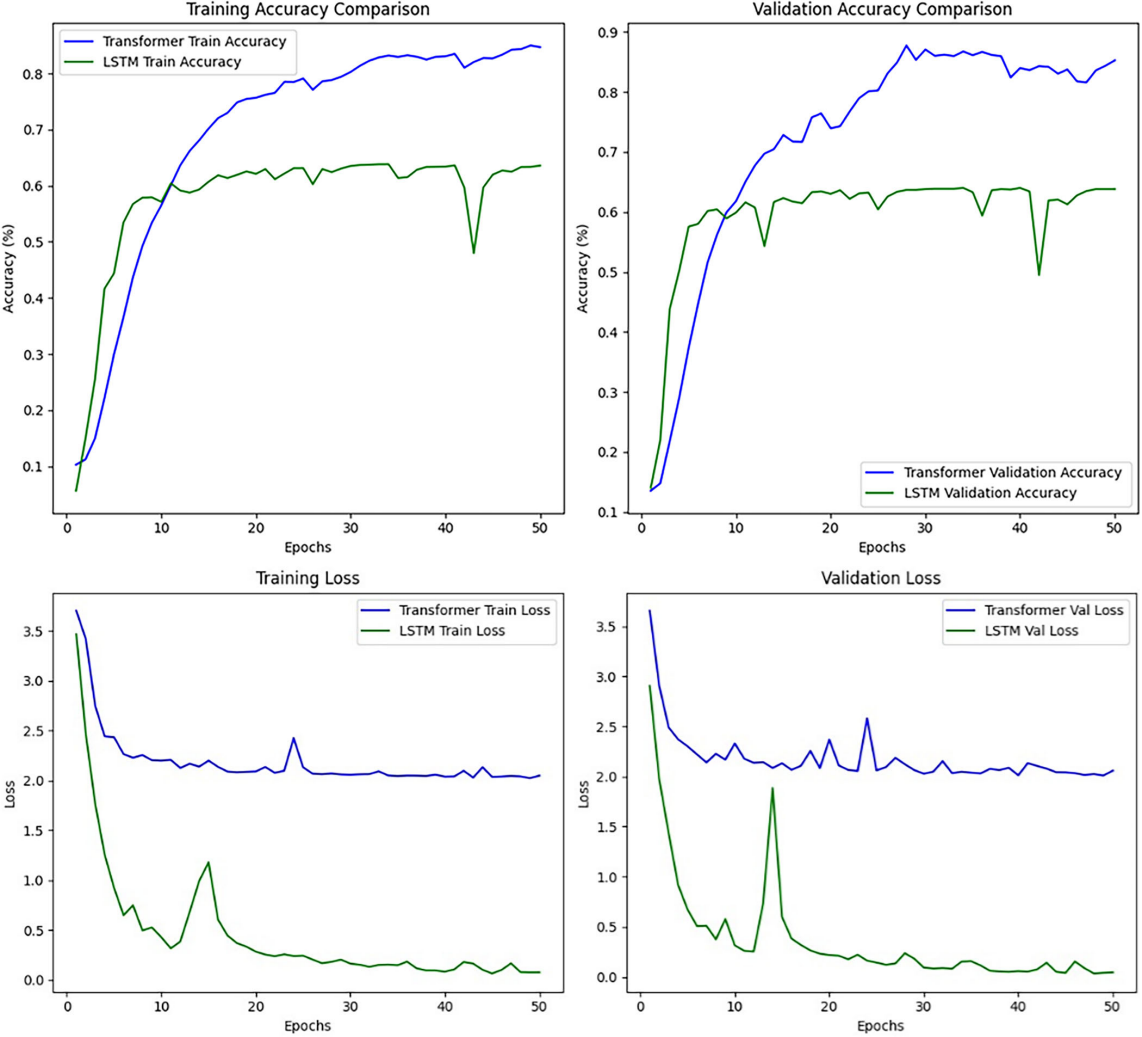
Training and validation accuracy and loss comparison of transformer and LSTM models.

Finally, the performance of our symptom‐based disease prediction model with an encoder‐only transformer architecture integrated into the Virtual Vita platform is presented in this section. Encoder‐only transformers are used in understanding all types of tasks as they only focus on features of input data. Many models, such as the Bidirectional Encoder Representations from Transformers (BERT) model, play a powerful role in natural language understanding applications, including text classification, named entity recognition, and sentiment analysis [23]. The proposed transformer model works in both ways, and therefore reduces dependency on the future tokens, making it effective for semantic analysis. Encoder‐only architectures are most beneficial in areas that require increased realism, new ways of practicing interpretability, further domain specialization, and novel methods of pretraining for several downstream applications. The model was trained over 50 epochs and its accuracy and loss results are shown below in Figure 3.

As expected, the transformer‐based model exhibited strong performance in predicting diseases. The model exhibited the ability to learn and generalize effectively over the data set. Over 50 epochs, the performance of LSTM and transformer‐based models were compared across training and validation metrics and the four graphs illustrate their performance. Training accuracy comparison (in the top left graph) shows that, although both models increased in accuracy, the transformer (blue line) model exhibited better performance than the LSTM (green line) model, with a peak accuracy rate of approximately 85%, whereas that of the LSTM model reached approximately 65% with significant fluctuations. Unlike the other two methods, validation accuracy comparison, also shown in the top right, showed that the transformer model outperformed the LSTM model with validation accuracy of approximately 90%, followed by the LSTM with accuracy of approximately 65%, with small fluctuations in both cases. The training loss comparison in the bottom left graph shows that the LSTM model exhibited a more rapid decrease in training loss compared with the transformer model, but exhibited some instability suggesting an over‐fitting or learning problem. In contrast, the transformer model's loss reduction was steady and slower. These observations are in accord with the results of the validation loss comparison shown in the bottom right graph, which indicated that the transformer model generated more stable results but higher losses, whereas the LSTM exhibited lower losses and sharper variations. Overall, the results revealed that the transformer model achieved higher accuracy and stability, whereas the LSTM model converged faster, though with less reliable loss progression, potentially suggesting that overfitting or generalization was implicitly enforced (Figure 3).

**Figure 4:**
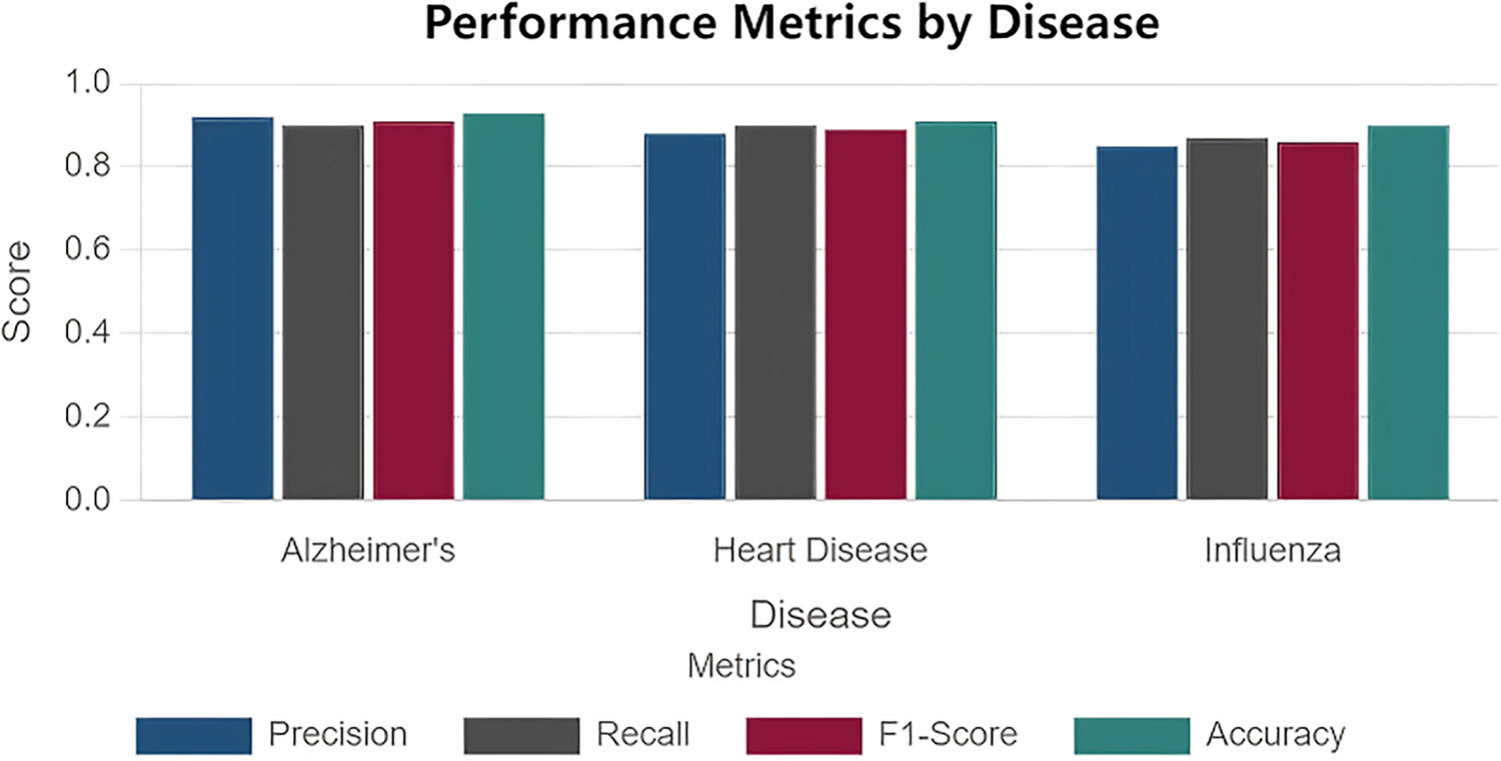
Classification results of our model for Alzheimer's disease, heart disease, and influenza.

The bar graph of Figure 4 shows the classification results of our model for Alzheimer's disease, heart disease, and influenza, showing precision, recall, F1 score and accuracy. Heterogeneity across the scores (between diseases) was small and most scores were consistently very high (> 0.9). Accordingly, the model's performance was consistently high for all diseases. These results indicate that the model was robust and reliable in making accurate and suitable predictions with various diseases.

**Figure 5:**
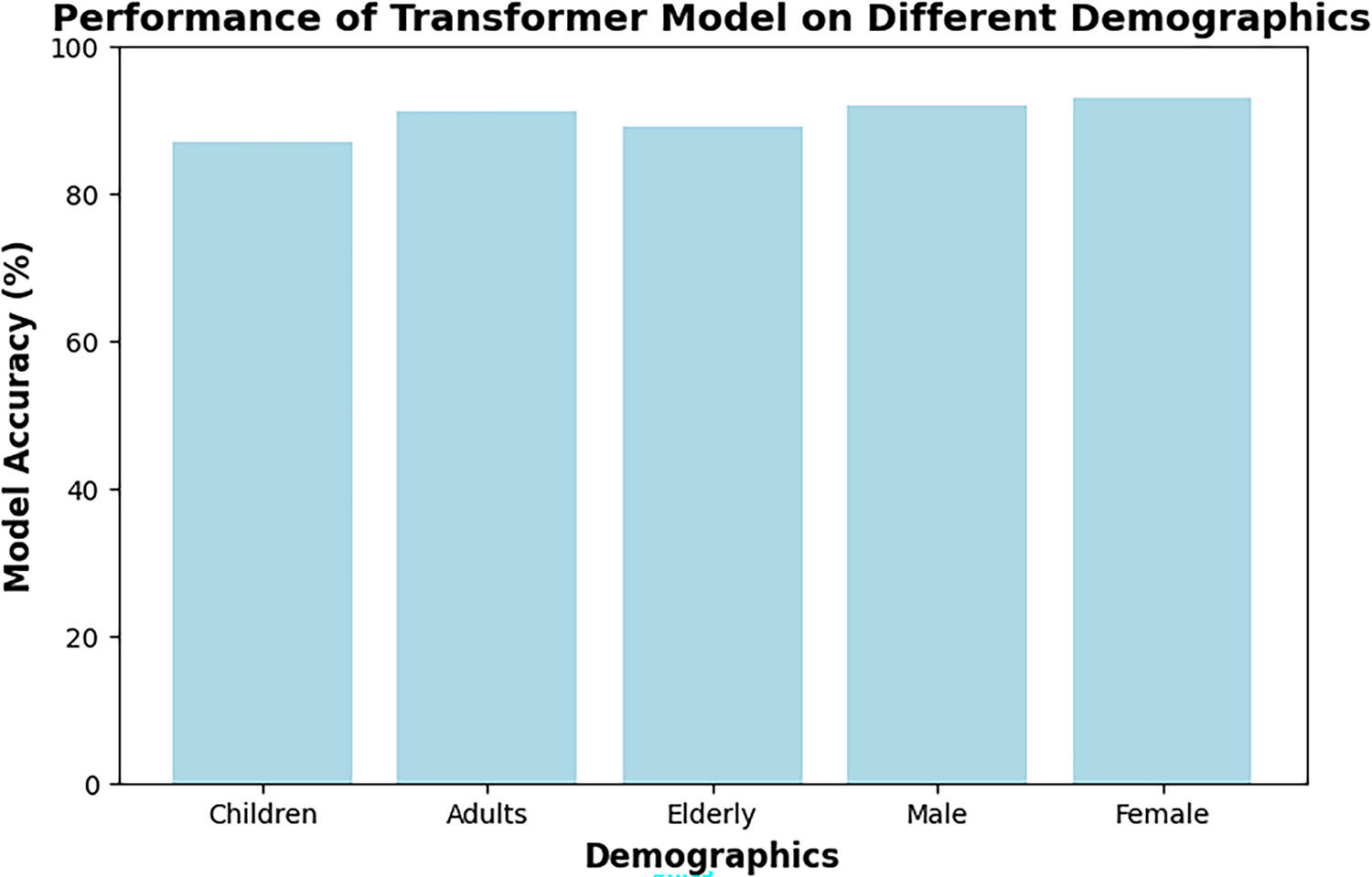
Performance of prediction model for different demographics.

To test the effectiveness of our prediction model, we evaluated its performance Disease Diagnosis Data set [12]. The results revealed strong evidence that the model was able to predict disease outcomes with high accuracy over multiple demographic groups as shown in Figure 5, with accuracy rates consistently above 80%. This finding suggests that the model is capable of recognizing the relationship pattern between symptoms and diseases, and can serve as a source of predictive healthcare analytics. For both gender inclusive healthcare predictions, this model also performs very well, ensuring its reliability. These findings suggest that with an extensive amount of medical data, a robust transformer‐based model can achieve accurate and unbiased disease predictions in real‐world healthcare scenarios.

**Figure 6:**
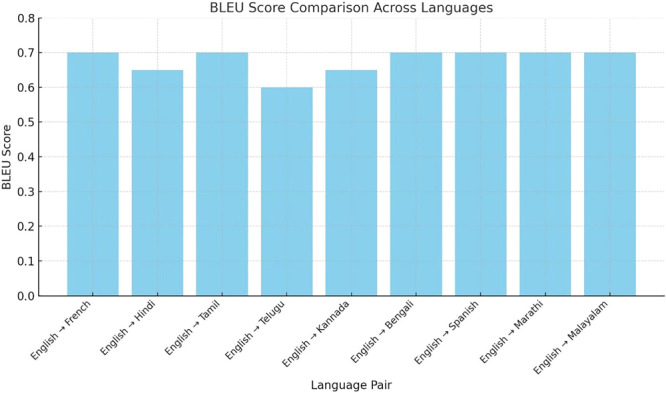
Graph of BLEU score comparisons across multiple languages.

The model was validated using BLEU score for its multilingual function, supporting English, Hindi, Spanish, German, French, Punjabi, Marathi, Telugu, Bengali, Tamil, Kannada, Malayalam, Gujarati, and Oriya. The BLEU scores represent a measurement of the model's translation ability from English to multiple languages. It can be seen in Figure 6 that English to French achieved the highest BLEU score 0.7, reflecting better quality of translation. Moderate BLEU scores of 0.42 for Hindi, 0.79 for Tamil, 0.39 for Telugu, and Kannada suggested moderately consistent performance. Other scores included 0.68 for Bengali, 0.7 for Spanish, 0.68 for Marathi, and 0.68 for Malayalam. BLEU score differences may indicate the quantity and quality of available data for each language pair. The high score for the French translation may have occurred because more standardized translation data or more robust linguistic resources were available for French, increasing the score (Figure 6).

The variation of BLEU scores across languages was investigated, revealing that variations were caused by linguistic, structural and evaluation specific factors. For example, English, Hindi, Marathi, and Tamil do not just differ in their grammar and syntax, but also in the word order leading to mismatched n‐grams in BLEU scoring. This also demonstrates that rich morphological languages such as Kannada, Malayalam, and Tamil present difficulties in tokenization patterns for which a language‐specific preprocessing pipeline is adopted. In the current study, unique linguistic features were accounted with optimal tokenization techniques.

## 4 Discussion

In the current study, LSTM‐ and transformer‐based architectures were evaluated. The results revealed that, in symptom‐based disease risk assessment, a transformer model performed better than a classical sequential LSTM model. Experimental results indicated that the encoder‐only transformer model significantly outperformed the LSTM model. The ability of the transformer model to capture relationships between symptoms using a self‐attention mechanism was key to extracting the relevant features pertinent to disease diagnosis and helped to improve generalization.

The proposed system supports a multilingual chatbot translation task across 14 languages [24]. A comparison of BLEU scores revealed that English to French translation had the highest performance with a BLEU score of 0.7, whereas Telugu translation exhibited the lowest performance with a BLEU score of 0.39 because of linguistic and data constraints. English and Hindi dominated the data set, followed by Bengali and Gujarati. This technology may improve access to the digital world, and assist healthcare professionals, but must be integrated with care to maintain values such as empathy, fairness, and professional judgment.

AI technologies like transformer‐based models could be helpful for focusing the healthcare industry on precision and accessibility over time, although ongoing research and development is still required to ensure that technological progress is balanced with human inputs involving care and compassion [25]. Existing limitations of virtual assistants should be acknowledged, and these methods should be considered as supplements not replacements for the power of human expertise while focusing on more inclusive and productive ways to serve patients and providers alike [19].

## 5 Conclusion

According to our findings, the encoder‐only Transformer models can outperform LSTMs in terms of symptom‐based disease prediction due to comprising high‐order interactions of symptoms. Yet, low BLEU scores among the underrepresented languages such as Telugu point to the lack of data diversity. Although the multilingual chatbot has a potential, the safety and effectiveness of its application in clinical reality remains to be confirmed which is part of future work. The suggested system provides overall a step toward smarter and more accessible healthcare assistance, though judicious integration with human supervision is still necessary.

## Author Contributions

**Geetika Munjal:** supervision (equal), writing – review and editing (lead). **Piyush Agarwal:** conceptualization (equal), data curation (equal). **Lakshay Goyal:** formal analysis (equal), resources (equal), visualization (equal). **Nandy Samiran:** visualization (supporting), writing – original draft (supporting).

## Ethics Statement

The authors have nothing to report.

## Consent

The authors have nothing to report.

## Conflicts of Interest

The authors declare no conflicts of interest.

## Data Availability

The data set is taken for public repository Kaggle (https://www.kaggle.com/datasets/pasindueranga/disease-prediction-based-on-symptoms. http://www.kaggle.com/datasets/s3programmer/disease-diagnosis-dataset).
